# Development of an Immune-Related Risk Signature in Patients with Bladder Urothelial Carcinoma

**DOI:** 10.1155/2020/5848493

**Published:** 2020-08-20

**Authors:** Yaofei Jiang, Yibing Wang, Cong Li, Zhenhong Zou, Bo Liang

**Affiliations:** ^1^Department of General Surgery, Second Affiliated Hospital of Nanchang University, 1 Minde Road, Nanchang, 330006 Jiangxi, China; ^2^Department of Radiation Oncology and Medical Oncology, Zhongnan Hospital, Wuhan University, Wuhan, China; ^3^Department of Emergency, Second Affiliated Hospital of Nanchang University, 1 Minde Road, Nanchang, 330006 Jiangxi, China

## Abstract

With recent advances in immunooncology and tumor microenvironment, the treatment landscape of bladder urothelial carcinoma has been changing dramatically. We aim to construct an immune gene-related signature which can predict BLCA patients' overall survival. Transcriptomic data of BLCA patients was downloaded from The Cancer Genome Atlas database, and immune-related genes were downloaded from the Immunology Database and Analysis Portal database. Prognostic immune-related genes were identified. We then constructed and validated an immune gene-related signature. Tumor-related transcription factors were downloaded from the Cistrome database, and a network between them and prognostic immune-related genes was generated. Cox's proportional hazards model and the Kaplan–Meier survival analysis were performed to assess our signature's prognostic ability. Relationship between the signature and patients' clinicopathologic features was then explored to validate its clinical value. We further downloaded concentration of six types of immune cells from the Tumor Immune Estimation Resource database to explore immune-related potential mechanisms of the signature.

## 1. Introduction

Bladder cancer is the most common cancer in the urinary tract and the ninth most common cancer worldwide, with 549393 new cases and 199922 deaths in 2018 [1, 2]. There are 80470 new cases and 17670 deaths expected in the USA in 2019 according to the latest epidemiological data on bladder cancer [3]. Bladder urothelial carcinoma (BLCA) is the most common histopathological type of bladder cancer [3, 4]. About a quarter of BLCA patients are muscle invasive bladder cancer (MIBC) and the rest are nonmuscle invasive bladder cancer (NMIBC) [5–7]. Surgery is one of the main treatments for BLCA. However, more than 30% of BLCA patients will experience tumor recurrence after surgery, which eventually bring bladder cancer 13^th^ most common cause of cancer death [2, 8]. With high recurrence and metastasis feature, BLCA patients suffer from great mental and economic pressure, as well as severe physical pain. Therefore, further exploration of BLCA patients' diagnosis and treatment is urgently necessary.

There is an upsurge of interest in tumor immune microenvironment (TIM) in recent years. With either immune suppressor or immune promoter ability, a variety of immune cells make up TIM, which can limit T cells' accumulation at where cancer cells locate [9]. Interestingly, immunotherapy has been playing an important role in the treatment of BLCA for more than 40 years and intravesical Bacillus Calmette-Guerin (BCG) remains to be the most efficacious intravesical medicament for NMIBC [10]. Although there are no available results yet focusing on the efficacy of immune checkpoint inhibitors (CPIs) in patients with MIBC, many trials in terms of immunotherapy in the neoadjuvant or adjuvant therapy for BLCA are currently recruiting and ongoing [10–13]. Also, encouraged by the promising results of several pivotal trials, various large trials with regard to the approval of the CPIs (avelumab, nivolumab, pembrolizumab, and atezolizumab) have been set up to explore the safety and efficacy of them in the treatment for patients of metastatic BLCA [14–17]. Balar et al.'s study demonstrated the efficacy and safety of first-line atezolizumab utilization for cisplatin ineligible patients with metastatic and locally advanced urothelial cancer, as well as the efficacy and safety of pembrolizumab for these patients [18, 19]. Nevertheless, there has been no signature based on immune-related genes with prognostic ability which can assess the individual immune status.

In this study, transcriptome data of BLCA patients were downloaded from The Cancer Genome Atlas (TCGA). We constructed and validated an immune signature which consists of 10 immune-related genes. Then, we evaluated the association between this signature and patients' survival outcome and clinical features. In addition, a network of tumor-related transcription factors (TFs) and immune-related genes was developed to further figure out the potential mechanisms of this signature. Finally, we explore the association between the signature and immune cells' infiltration.

## 2. Materials and Methods

### 2.1. BLCA Patient Information and Immune-Related Genes Predicting Prognosis Risk

The transcriptomic data and matching clinical information of BLCA patients were downloaded from The Cancer Genome Atlas (TCGA) data portal (up to September 18, 2019; https://portal.gdc.cancer.gov). Clinical information of BLCA patients were also downloaded, including age, gender, grade, stage, and TNM classification. The comprehensive list of a total of 2498 immune-related genes was obtained from the Immunology Database and Analysis Portal (ImmPort) database (https://immport.niaid.nih.gov) [20]. The Wilcoxon test was used to identify the differential gene between tumor and normal tissues. The genes with ∣Log fold change (LogFC) | >1 were thought to be differential gene. “Survival” R package and Cox analysis were used to find the differential immune-related gene with prognostic ability (*p* < 0.01).

### 2.2. Network between Immune-Related Genes and Transcription Factors

The list of a total of 318 TFs was attained from the Cistrome database (http://cistrome.org/CistromeCancer/). Correlation analysis between differential immune-related genes predicting prognosis and TFs was done using cor.test. Cytoscape 3.6.1 was used for network graph drawing.

### 2.3. Immune-Related Risk Signature Construction and Performance Assessment

The Cox proportional hazards model was performed to identify the best immune-related gene model for predicting the prognosis in BLCA patients [21]. All patients were divided into high- and low-risk groups according to median immune-related risk score based on the model. The Kaplan–Meier (K-M) survival curve was performed to exhibit the overall survival (OS) of the two groups. ROC curve was performed and the area under the curve (AUC) was calculated to evaluate the prognostic capability of the immune-related risk signature [22]. The univariate and multivariate analyses of survival were put up for both clinicopathologic features and immune signature. Correlation analysis between genes involved in the signature and clinicopathologic features was also performed.

### 2.4. Correlation Analysis of the Signature and Immune Cells

The concentration of six types of immune cells in all samples in TCGA database, including neutrophil, macrophage, dendritic cell, B cell, CD4 T cell, and CD8 T cell, was downloaded from the Tumor Immune Estimation Resource (TIMER) database (http://cistrome.dfci.harvard.edu/TIMER/). Correlation analysis between six types of immune cells and risk score of BLCA patients were then conducted.

### 2.5. Statistical Analysis

Student's *t* test was conducted to make statistical comparison. Heatmaps were generated using “Pheatmap” R package. The Kaplan–Meier (K-M) survival curves were generated utilizing the “survival” R package. “SurvivalROC” R package was used to produce ROC curve. All of our analysis was conducted using the R software version 3.5.0 (https://www.r-project.org/). A *p* value < 0.05 was thought to be statistically significant.

## 3. Results

### 3.1. Identification of Prognostic Immune-Related Genes and Transcription Factors and Network Development

**Figure 1:**
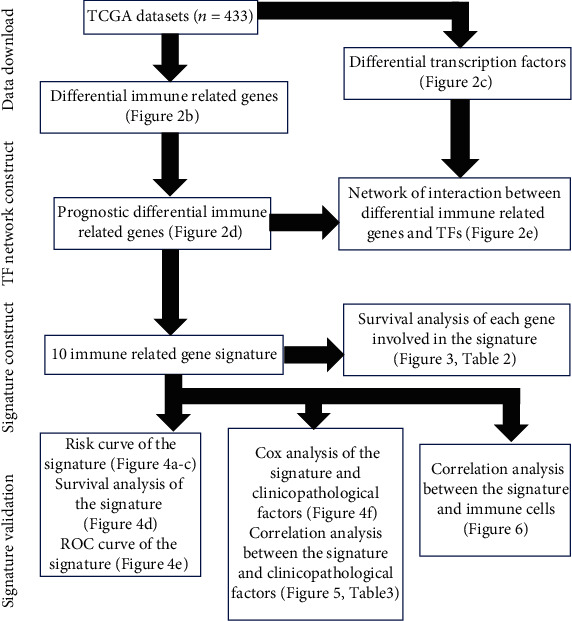
The workflow demonstrating the schematic overview of the project.

**Figure 2:**
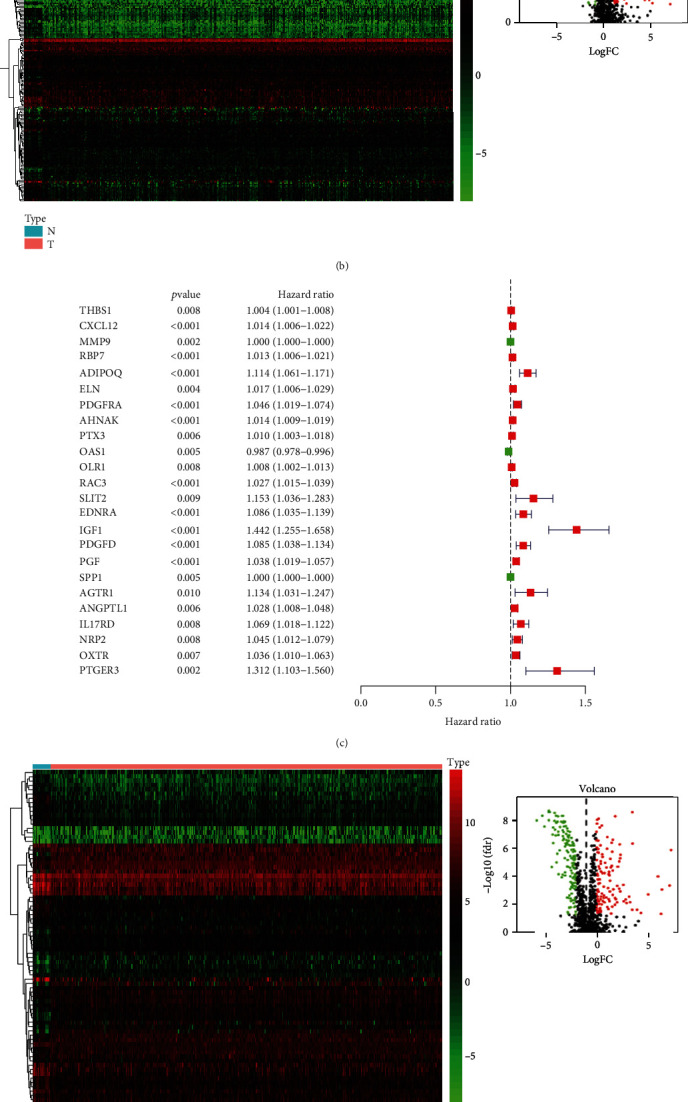
Heatmap and volcano of (a) differential genes, (b) differential immune-related genes, and (c) differential TFs between tumor and normal samples. Forest plots of hazard ratios of 24 immune-related genes with prognostic ability (d). Network of interaction between differential immune-related genes and TFs (e).

To make our study clearer, a workflow of it is shown in Figure 1. A total of 433 BLCA patients' RNA-seq data were collected from TCGA database. The patients' clinical and survival information were summarized in Table 1. A total of 4876 differential genes between tumor and normal samples, with 1423 downexpression genes and 3453 upexpression genes, were identified. The heatmap and volcano figure are shown in Figure 2(a). Among these differential genes, 120 upexpression and 140 downexpression immune-related genes were recognized (Figure 2(b)). After univariate analysis (*p* < 0.01), 24 immune-related genes with prognostic ability were identified, and the forest plot is shown in Figure 2(c). As shown in Figure 2(d), 41 upexpression and 36 downexpression TF genes were recognized from the differential genes. Then, correction analysis was down for the 24 immune-related genes and differential TF genes using cor.test (corFilter = 0.4). The interactions between TFs and immune-related genes are graphically demonstrated in Figure 2(e).

### 3.2. Construction and Validation of the Immune-Related Risk Signature

**Figure 3:**
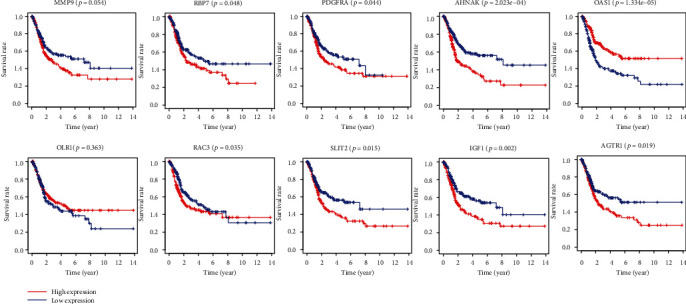
The K-M analysis of the 10 immune-related genes used to construct the immune-related risk signature for BLCA, including RBP7, PDGFRA, AHNAK, RAC3, IGF1, AGTR1, SLIT2, OAS1, MMP9, and OLR1.

To construct an immune-related risk signature, we used “survival” R package to build proportional hazards model. Eventually, 10 genes were elected from the 24 immune-related genes to form an immune-related risk signature, including 8 relatively high-expression genes (MMP9, RBP7, PDGFRA, AHNAK, OLR1, RAC3, IGF1, and AGTR1) and 2 relatively low-expression genes (OAS1, SLIT2). The multivariate analysis of the 10 genes is shown in Table 2, and the K-M analysis of each gene is exhibited in Figure 3. To validate our signature, the risk score of patients was evaluated according to the coefficient value of the 10 genes as follows: risk score = (0.0003617∗MMP9) + (0.0096326∗RBP7) + (0.0399101∗PDGFRA) + (0.014035∗AHNAK) + (−0.008116∗OAS1) + (0.0072663∗OLR1) + (0.0297334∗RAC3) + (−0.209438∗SLIT2) + (0.2684786∗IGF1) + (0.1416244∗AGTR1).

**Figure 4:**
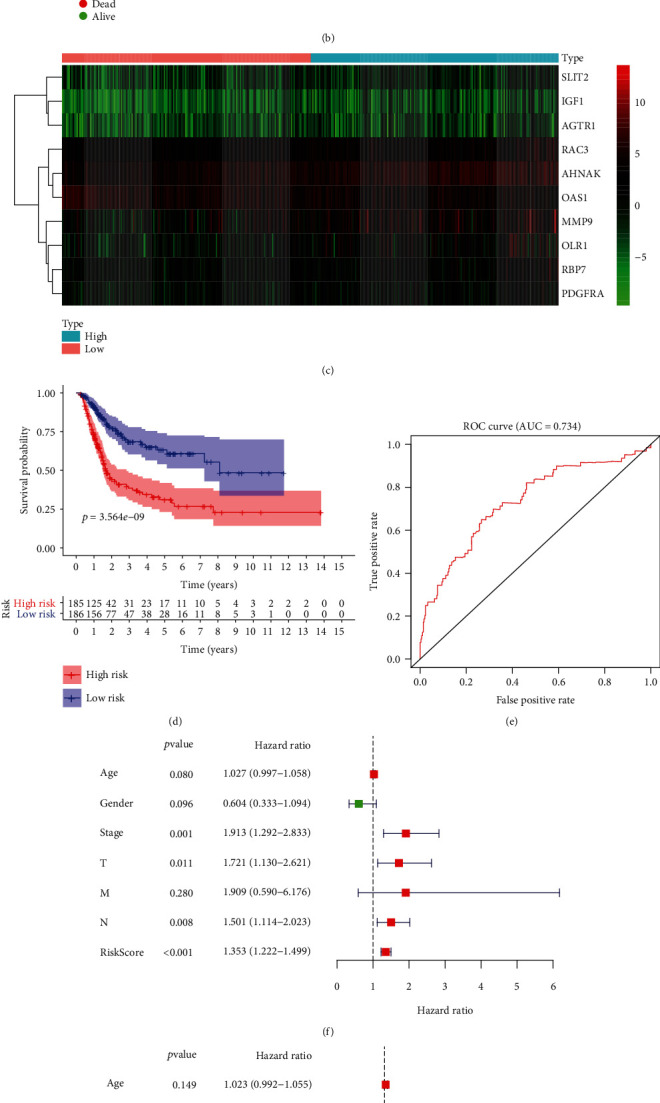
Heatmap of the signature consisting of 10 immune-related genes used to construct the immune-related risk signature for BLCA (a–c). The Kaplan–Meier survival analysis of the signature (d). ROC curve analysis of the signature (e). The Cox analysis of the signature and clinicopathological factors: the univariate Cox analysis (f); the multivariate Cox analysis (g).

Then, the patients were divided into the high-risk group or the low-risk group according to median immune-related risk score, as demonstrated in Figures 4(a)–4(c). The K-M analysis was done and results showed that patients with high risk had a poor overall survival (OS) compared with those with low risk (Figure 4(d), *p* < 0.001). After that, the ROC curve analysis of the signature showed the promising predictive value of it for BLCA patients' survival (AUC = 0.734, Figure 4(e)). Furthermore, as shown in Figure 4(f), the univariate Cox analysis revealed significant association between the signature and BLCA patients' OS, as well as stage and T and N classification. Multivariate Cox analysis further demonstrated that our immune-related signature could serve as an independent predictor of patients' OS (hazard ratio (HR) = 1.303, 95% confidence intervals (95% CI) 1.145 to 1.483, *p* < 0.001). The results showed that the signature could be an independent predictor for patients' OS, which indicated that our signature has a strong prognostic ability.

### 3.3. Correlation of the Immune-Related Risk Signature with Clinicopathologic Features

**Figure 5:**
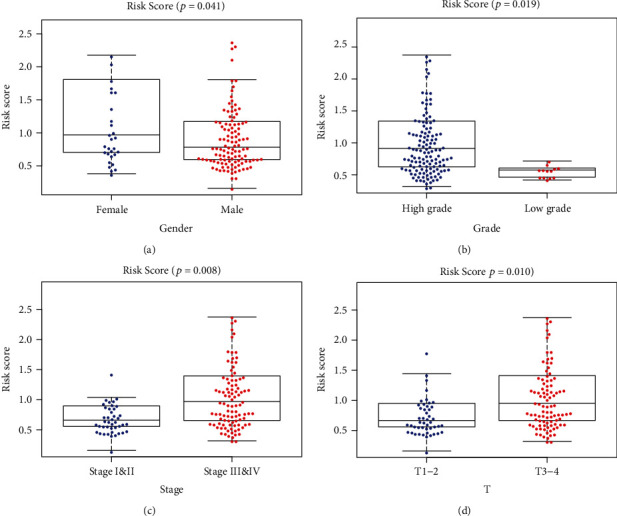
The scatter plot of the relationship between the signature and (a) gender, (b) grade, (c) staging, and (d) T stage.

To further validate the clinical value of the signature in BLCA patients, we evaluated the relationship between the 10-gene immune signature and clinicopathologic features. Patients with high risk tend to be male and have advanced grade, stage, and T classification (Figure 5). Eight genes except OLR1 and AGTR1 were significantly associated with patients' grade. Six genes except MMP9, OLR1, RAC3, and ATTR1 were significantly associated with patients' stage. The other association of the genes and clinicopathologic factors is also demonstrated in Table 3.

### 3.4. Association between the Immune-Related Risk Signature and Immune Cells

**Figure 6:**
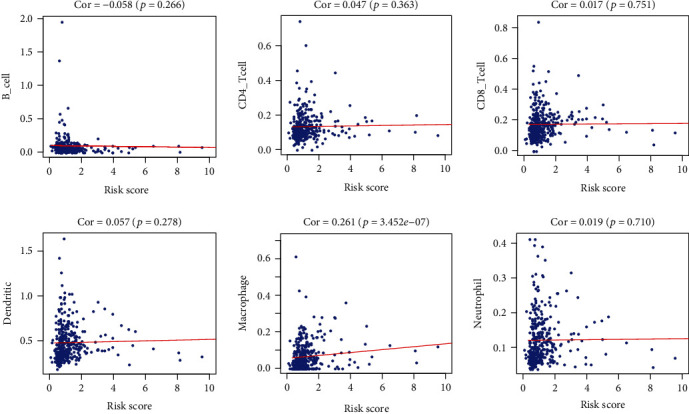
The scatter plot of the relationship between the signature and immune cells.

In order to further explore immune-related potential mechanisms of the signature, we appraised the association of our signature and six types of immune cells, including neutrophil, macrophage, dendritic cell, B cell, CD4 T cell, and CD8 T cell. We found that high-risk group patients tended to have more macrophage cell infiltration and no significant change in other five kinds of immune cells (*p* < 0.05, Figure 6).

## 4. Discussion

It is believed that BLCA is a complex and intractable disease with high morbidity and mortality which necessitates long-term monitoring [23]. Therefore, both diagnosis ability and treatment of BLCA patients are urgently needed to be improved. With the dramatic development of immunooncology and tumor microenvironment in recent years, immune environment is conformed to play more and more important role in the development of cancer [24]. Therefore, it is essential to explore an immune-related signature which can not only provide immune-related biomarkers for BLCA patients' prognosis but also may serve as a momentous reference in immunotherapy.

In this study, we constructed a stable immune-related signature consisted of 10 immune genes with prognostic ability for BLCA patients utilizing TCGA BLCA dataset. The signature may represent the status of BLCA patients' TIM and prognosis and provide potential targets for immunotherapy. We then evaluated the correlation of the signature and patients' OS and clinicopathological factors to validate its clinical values. The results showed that patients with high immune risk have a poor OS and tend to be male and have advanced grade, staging, and T stage. These suggest that the TIM of patients with high immune risk can promote the development and therefore lead to advanced stage and grade. In addition, multivariate analysis further confirms that the signature can be an independent predictor for BLCA patients. Therefore, the immune-related signature can not only predict BLCA patients' survival outcome but also indicate the disease progression.

In order to make out the molecular mechanisms of this immune-related signature, we investigated the genes involved in the signature. Interestingly, IGF1 was the most significant immune gene in the univariate analysis of the 10 immune-related genes' signature (HR = 1.3080, *p* = 0.0016). Insulin-like growth factors (IGFs) are known regulator of energy metabolism and growth. IGF1 belongs to the IGF family and is a part of the metabolic system that includes insulin and two adipocytokines (leptin and adiponectin) [25–28]. More studies reported the role of IGF1 in the development of BLCA. Dunn et al. reported that reduced serum IGF1 would suppress bladder tumor progression in p53-deficient mice [29]. IGF1 can block apoptosis in human bladder cancer cells and increase circulating IGF1, thereby augmenting risk of BLCA patients [30, 31]. Long et al. reported that increased IGF1 can promote cisplatin resistance in bladder cancer cells. Therefore, we suggest that IGF1 may bridge metabolic system and immune oncology. In the signature, SLIT2 is another gene attracting our attention. Sherchan et al. reported that recombinant SLIT2 attenuates neuroinflammation by inhibiting peripheral immune cell infiltration [32]. Similar results were also found in other studies. Chaturvedi et al. reported that SLIT2 can prevent neutrophil recruitment and Guan et al.'s study showed that SLIT2 can inhibit the development of immune responses [33, 34]. More interestingly, the interaction network showed that upstream transcription factor GATA6 and NFATC1 can both bind to IGF1 and SLIT2 which indicated the potential importance of TGF1 and SLIT2 in immune responses. Furthermore, macrophages tend to infiltrate in high-risk group patients. All these results reflect the importance of our immune-related signature in the BLCA microenvironment.

Taken together, based on our knowledge, this is the first study that identified the 10 immune-related genes' signature which can not only be an independent predictor for BLCA patients' survival outcome but also may provide novel targets for immunotherapy for them. Another advantage of this study was using massive data from TCGA database to build and validate the immune-related signature. Nonetheless, several limitations still exist. Firstly, this is a retrospective study with relatively limited samples. Then, we did not validate the signature with external data although we have verified the validity and stability of the signature in various aspects. Finally, the signature should be tested for its predictive ability in the clinical environment and the 10 immune-related genes also need to be further explored.

## 5. Conclusions

In conclusion, the present study constructed an immune-related signature containing a total of 10 immune-related genes, including MMP9, RBP7, PDGFRA, AHNAK, OAS1, OLR1, RAC3, SLIT2, IGF1, and AGTR1. This signature expressed a strong prognostic ability and served as an independent predictor for BLCA patients' survival and was significantly associated with gender, stage, grade, and T classification. High-risk score group patients presented more macrophage cell infiltration. The findings of this study provide potential novel targets and may promote individualized immunotherapy.

## Figures and Tables

**Table 1:** Summary of clinical and pathological features of BLCA patients.

Variable	Number of patients (*n* = 412)	Variable	Number of patients (*n* = 412)
*Age*		*Gender*	
≤65	162	Male	304
>65	250	Female	108
*State*		*Grade*	
Alive	253	High	388
Dead	159	Low	21
		Unknown	3
*Stage*		*T stage*	
Stage I	2	T1	3
Stage II	131	T2	121
Stage III	141	T3	196
Stage IV	136	T4	59
Unknown	2	Unknown	33
*N stage*		*M stage*	
N0	239	M0	196
N1	167	M1	11
Unknown	6	Unknown	205

**Table 2:** Multivariate Cox analysis for overall survival of 10 immune-related genes involved in the signature.

id	coef	HR	HR.95L	HR.95H	*p* value
S100B	0.030657	1.031132	0.999272	1.064006	0.055555
MMP9	0.000257	1.000257	1.000065	1.00045	0.008723
VAV2	0.03279	1.033334	1.010431	1.056756	0.004138
TYMP	0.006076	1.006095	1.003407	1.008789	8.48*E*-06
ARTN	0.043398	1.044353	0.992476	1.098942	0.095033
BDNF	0.567795	1.764373	1.191978	2.611635	0.004545
DKK1	0.002473	1.002476	0.999753	1.005207	0.074803
AVPR2	0.629915	1.877451	1.320914	2.668471	0.000446

CI: confidence interval; HR: hazard ratio.

**Table 3:** The relationship between the signature and clinical features.

id	Age	Gender	Grade	Stage	T	M	N
MMP9	0.005 (0.996)	0.569 (0.571)	5.384 (2.875*e*-07)	−1.611 (0.110)	−1.966 (0.051)	−1.098 (0.319)	−0.592 (0.555)
RBP7	−0.7 (0.485)	1.125 (0.268)	2.232 (0.027)	−2.055 (0.042)	−1.859 (0.066)	−1.316 (0.245)	−1.611 (0.114)
PDGFRA	−0.422 (0.674)	0.778 (0.442)	3.048 (0.004)	−3.046 (0.003)	−3.008 (0.003)	−0.481 (0.647)	−1.242 (0.218)
AHNAK	−0.899 (0.370)	1.378 (0.176)	5.605 (7.957*e*-07)	−3.667 (3.488*e*-04)	−3.898 (1.461*e*-04)	0.926 (0.389)	−2.267 (0.026)
OAS1	2.082 (0.039)	−2.634 (0.010)	−2.181 (0.045)	2.19 (0.032)	2.18 (0.032)	2.913 (0.023)	1.74 (0.085)
OLR1	0.702 (0.484)	−0.098 (0.922)	1.415 (0.170)	−1.059 (0.292)	−1.021 (0.309)	2.971 (0.005)	−0.393 (0.695)
RAC3	−0.491 (0.624)	0.868 (0.390)	2.724 (0.009)	−0.472 (0.638)	0.142 (0.887)	−1.139 (0.306)	−1.763 (0.083)
SLIT2	−0.968 (0.335)	1.006 (0.320)	3.448 (0.001)	−3.546 (5.295*e*-04)	−3.545 (5.237*e*-04)	−1.724 (0.144)	−1.937 (0.057)
IGF1	0.152 (0.879)	0.776 (0.442)	2.909 (0.004)	−3.54 (5.794*e*-04)	−3.087 (0.003)	−0.981 (0.365)	−1.126 (0.264)
AGTR1	−1.482 (0.141)	0.54 (0.592)	−0.795 (0.439)	0.37 (0.712)	0.427 (0.670)	0.032 (0.976)	0.719 (0.473)
riskScore	−0.297 (0.767)	2.126 (0.041)	2.479 (0.019)	−2.683 (0.008)	−2.595 (0.010)	−0.931 (0.394)	−1.806 (0.075)

## Data Availability

The data that supported the findings of this study were derived from The Cancer Genome Atlas (TCGA) data portal (https://portal.gdc.cancer.gov), the Immunology Database and Analysis Portal (ImmPort) database (https://immport.niaid.nih.gov), and the Cistrome database (http://cistrome.org/CistromeCancer/). It was also available from the corresponding author.
